# Differential pattern of glycogen accumulation after protein phosphatase 1 glycogen-targeting subunit PPP1R6 overexpression, compared to PPP1R3C and PPP1R3A, in skeletal muscle cells

**DOI:** 10.1186/1471-2091-12-57

**Published:** 2011-11-04

**Authors:** Marta Montori-Grau, Maria Guitart, Cèlia García-Martínez, Anna Orozco, Anna Maria Gómez-Foix

**Affiliations:** 1 CIBER de Diabetes y Enfermedades Metabólicas Asociadas (CIBERDEM) Pg de la Bonanova 69, 6a planta, 08017-Barcelona, Spain; 2 Departament de Bioquímica i Biologia Molecular, IBUB, Facultat de Biologia, Universitat de Barcelona, Diagonal 643, 08028-Barcelona, Spain; 3 Departament de Patologia i Terapèutica Experimental, Unitat organitzativa Bellvitge, Universitat de Barcelona, Pavelló de Govern, Pl. 5a, Feixa Llarga, S/N, 08907-Hospitalet de Llobregat, Spain

## Abstract

**Background:**

PPP1R6 is a protein phosphatase 1 glycogen-targeting subunit (PP1-GTS) abundant in skeletal muscle with an undefined metabolic control role. Here PPP1R6 effects on myotube glycogen metabolism, particle size and subcellular distribution are examined and compared with PPP1R3C/PTG and PPP1R3A/G_M_.

**Results:**

PPP1R6 overexpression activates glycogen synthase (GS), reduces its phosphorylation at Ser-641/0 and increases the extracted and cytochemically-stained glycogen content, less than PTG but more than G_M_. PPP1R6 does not change glycogen phosphorylase activity. All tested PP1-GTS-cells have more glycogen particles than controls as found by electron microscopy of myotube sections. Glycogen particle size is distributed for all cell-types in a continuous range, but PPP1R6 forms smaller particles (mean diameter 14.4 nm) than PTG (36.9 nm) and G_M _(28.3 nm) or those in control cells (29.2 nm). Both PPP1R6- and G_M_-derived glycogen particles are in cytosol associated with cellular structures; PTG-derived glycogen is found in membrane- and organelle-devoid cytosolic glycogen-rich areas; and glycogen particles are dispersed in the cytosol in control cells. A tagged PPP1R6 protein at the C-terminus with EGFP shows a diffuse cytosol pattern in glucose-replete and -depleted cells and a punctuate pattern surrounding the nucleus in glucose-depleted cells, which colocates with RFP tagged with the Golgi targeting domain of β-1,4-galactosyltransferase, according to a computational prediction for PPP1R6 Golgi location.

**Conclusions:**

PPP1R6 exerts a powerful glycogenic effect in cultured muscle cells, more than G_M _and less than PTG. PPP1R6 protein translocates from a Golgi to cytosolic location in response to glucose. The molecular size and subcellular location of myotube glycogen particles is determined by the PPP1R6, PTG and G_M _scaffolding.

## Background

The main glycogen stores are confined to skeletal muscle and liver, although glycogen metabolizing machinery is present in many cell types. In the cell, glycogen is associated with protein complexes [1-3] that include glycogen-metabolizing enzymes such as the synthesizing enzyme GS, the hydrolyzing enzyme glycogen phosphorylase (GP), glycogenin, branching enzyme and debranching enzyme, and regulatory proteins, such as the protein phosphatase 1 (PP1) that directs the metabolism of the glycogen particle. These glycogen and protein complexes, in the form of particles or granules, were designated glycosomes [1,2].

PP1 is the only known serine/threonine phosphatase that dephosphorylates GS and GP and changes its activation state [4]. The PP1 catalytic subunit (PP1c) is targeted to the glycogen particles by glycogen-associated regulatory subunits, PP1-GTSs. PP1-GTSs constitute a family of proteins including PPP1R3A, PPP1R3B, PPP1R3C, PPP1R3D and PPP1R3E with low amino acid identity, which is characterized by its conserved PP1c binding motif (the RVXF motif) [5], a glycogen-binding domain [6,7] and a PP1-substrate binding domain [7]. PP1-GTSs display tissue- and specie-specific expression. In human skeletal muscle, several members of the PP1-GTS gene family are expressed: the muscle-specific PPP1R3A/G_M_/PP1G/R_GL _[8,9]; PPP1R3C/PTG/PPP1R5 [9,10] and PPP1R3B/G_L _[11], which are expressed mostly in muscle and liver; the relatively widespread isoform PPP1R3D/PPP1R6 [9]; and PPP1R3E, most abundant in skeletal muscle and heart tissue in humans [7]. In contrast, in rodent skeletal muscle, the PPP1R3B [11] and PPP1R3E [7] genes are not significantly expressed.

The human PPP1R6 subunit was identified by Armstrong et al. [9] in the search for sequences related to the rat G_L _and human PPP1R5 proteins in an expressed sequence tag database. These authors demonstrated that the PPP1R6 protein specifically binds to purified PP1 and is associated with glycogen in the glycogen-sarcovesicle fraction from rabbit skeletal muscle. They detected PPP1R6 transcripts in all human tissues examined including brain, placenta, lung, liver, kidney and pancreas. The highest levels were found in skeletal muscle and heart. In a later study, the PPP1R6 gene transcript was detected in several human cancer cell lines, including small cell and non-small cell lung cancers, colorectal, gastric and ovarian cancers, and adult normal lung, colorectal and ovarian tissues, but not normal gastric tissue [12]. Only one previous study has demonstrated a metabolic functional role of PPP1R6 in stimulating glycogen accumulation in Chinese hamster ovary cells stably transformed with the insulin receptor [13].

In contrast, the metabolic control role of G_M _and PTG in the muscle glycogen metabolism has been amply described. PP1G/R_GL_/G_M _regulates basal GS and GP activity ratio in skeletal muscle, as observed in G_M_/R_GL _knockout mice [14,15] and is essential for GS activation in response to exercise [14], but not necessary for activation of GS by insulin [15]. In cultured human myotubes, G_M _also regulates both GS and GP activities and the effect of activating GS is enhanced in glucose/glycogen-depleted cells [16]. PTG regulates basal GS activation, is not required for insulin stimulation of GS and does not affect GP activity in skeletal muscle of mice bearing a heterozygous deletion of the PTG gene [17]. In cultured human myotubes, PTG has a powerful activating effect on GS, which is not glucose-dependent, and on glycogenesis; whereas it does not affect GP activity [18]. In this cell system, the effects of G_L _have also been reported, i.e. that G_L _activates GS, regardless of glucose, and glycogenesis more powerfully than G_M_, and does not affect the GP activation state [19].

Even though PP1-GTSs are presumed to contribute to organizing the glycogen particle [2,3], the ability to target glycogen particles to a specific subcellular location has only been suggested for G_M_/R_GL_, as it contains a sarcoplasmic reticulum (SR) binding domain [8,20] and is found associated with non-junctional SR membranes, forming a complex with GS and PP1c in rabbit skeletal muscle [21].

Here we applied metabolic and glycogen cytochemical techniques to determine the glycogenic effect of PPP1R6; and used transmission electron microscopy to define the distribution and morphology of PPP1R6-formed glycogen particles in cultured skeletal muscle cells, in comparison with PTG and G_M_. We also provide information about the subcellular location of the PPP1R6 protein in cultured myotubes.

## Methods

### Muscle samples and culture

Muscle samples and myogenic cells came from a cryopreserved collection of human skeletal muscle biopsies described in [22] and obtained with written approval from the Ethics Committee of the Hospital Sant Joan de Déu (Barcelona, Spain) according to Spanish legislation at that time. Myotubes were derived from confluent myoblast cultures; immediately after the start of myoblast fusion, DMEM/M-199 medium (3:1) with 10% FBS (fetal bovine serum), 10 μg/ml insulin, 4 mM glutamine, 25 ng/ml fibroblast growth factor and 10 ng/ml epidermal growth factor was replaced by DMEM/M-199 medium (3:1) with 10% FBS and 10 μg/ml insulin, to further stimulate differentiation.

Mouse C2C12 myoblasts and 293 cells were grown in DMEM with 10% FBS. In confluent cultures, C2C12 cell fusion was stimulated by incubation in DMEM medium with 10% HS (horse serum). The C2C12-mtRFP cell line stably expressing the mitochondrial matrix-targeted (mt) red fluorescent protein (RFP) [23], kindly provided by Dr. A. Zorzano (Universitat de Barcelona, Spain), was cultured as the C2C12 cell line.

### RNA extraction, reverse transcription (RT) and real-time PCR

Total RNA was extracted from tissue samples as described elsewhere [22]. Total RNA from cultured cells was extracted with the RNeasy Minikit (Qiagen, Valencia, USA). Extracts were homogenized with a Polytron (Kynematica Polytron, Westbury, USA). A 0.5 μg portion of total RNA was retrotranscribed with TaqMan RT reagents from Applied Biosystems (Branchburg, USA) using random hexamers. Real-time PCR was performed in the ABI PRISM 7700 sequence detection system or LightCycler 480 SW 1.5 with the TaqMan universal PCR master mix or LightCycler 480 Probes Master, respectively, and probes for human *PPP1R3D *and rabbit *PPP1R3A *genes from Applied Biosystems. β2-microglobulin or 18S rRNA gene was used as the endogenous control to normalize the threshold cycle (Ct) or crossing point (Cp) for each probe assay. Relative gene expression was calculated as 2^-ΔCt ^and gene fold change was calculated by the 2^-ΔΔCt ^or 2^-ΔΔCp ^method. Mean values ± SEM are shown.

### Transduction with recombinant adenoviruses

Myotubes were used, 10 days after differentiation-induction for cultured human myotubes; or 5 days after, for C2C12 myotubes. Forty-eight hours before the experiments, human myotubes were depleted of insulin; and 24 h before, they were depleted of FBS. C2C12 cells were depleted of HS, 24 h before the experiments. Myotubes were transduced with adenoviruses at a multiplicity of infection of 20 for 4 h (human myotubes) or 2 h (C2C12 myotubes). The Ad-GFP adenovirus encoding the enhanced green fluorescent protein (EGFP) under the control of the CMV cytomegalovirus promoter, or the adenovirus Ad-lacZ, encoding the *E. coli *ß-galactosidase gene with a nuclear location signal [24], were used as controls. An adenovirus containing the cDNA of human PPP1R3D downstream of the CMV promoter was prepared. To do this, plasmid *IRAKp961M1391Q2 *from *RZPD *(imaGenes GmbH, Berlin, Germany), containing the cDNA of human PPP1R3D inserted into the *pBluescriptR *vector, was digested with *XhoI *and *BglII *to elicit the PPP1R3D cDNA band, which was ligated into the *Litmus 28i *vector (New England BioLabs Inc., Ipswich, USA) cut with the same restriction enzymes. Then, PPP1R3D cDNA was excised with *XbaI *and *SalI *and ligated into the pAdCMVIcPA vector (provided by Dr. C. García-Martínez) cut with the same restriction enzymes. Five μg of resulting plasmid were cotransfected with 10 μg of pJM17 into 293 cells to generate the adenoviral construct Ad-R6 [25]. Ad-G_M_, encoding rabbit G_M_, and Ad-PTG, encoding mouse PTG are described in [26]. Ad-MGP encoding rabbit muscle GP is described in [27].

### Enzyme activities and glycogen

To measure GS and GP activities, 100 μl of homogenization buffer consisting of 10 mM Tris/HCl (pH 7.0) 150 mM KF, 15 mM EDTA, 600 mM sucrose, 15 mM 2-mercaptoethanol, 17 μg/l leupeptin, 1 mM benzamidine and 1 mM PMSF was used in order to scrape the cell monolayers off the frozen plates prior to sonication. The resulting homogenates were used for the determination of enzyme activities. GP activity was determined by the incorporation of [U-^14^C]glucose 1-phosphate into glycogen in the absence or presence of the allosteric activator AMP (1 mM) [28]. GS activity was measured in the absence or presence of 10 mM glucose 6-phosphate as described in [29]. An aliquot of the homogenates was used to measure protein concentration with the Bio-Rad protein assay (Bio-Rad, Hercules, USA).

To assess glycogen synthesis, cells were incubated with 10 mM [U-^14^C]glucose (0.05 μCi/μmol). To isolate glycogen, cell monolayers were scraped into 100 μl of 30% KOH and homogenates were boiled for 15 min. An aliquot of the homogenates was used to measure protein concentration with the Pierce BCA protein assay kit (Thermo Scientific, Rockford, USA). Homogenates were spotted onto Whatman 3 MM paper; and glycogen was precipitated by immersing the papers in ice-cold 66% ethanol. When appropriate, radioactivity in dried papers was counted in a Beta-radiation counter or papers were incubated in 0.4 M acetate buffer (pH 4.8) with 25 units/ml alpha-amyloglucosidase (Sigma-Aldrich, Madrid, Spain) for 120 min at 37°C to hydrolyze glycogen. In the latter, glucose released was measured enzymatically with the Glucose kit from Biosystems (Barcelona, Spain).

Glycogen was stained cytochemically with the periodic acid/salicyloyl hydrazide (PA-SH) method. Briefly, C2C12 cells grown on coverslips were fixed with methanol at -20°C for 5 min. After rinsing three times with PBS and once with distilled water, half the samples were incubated with 0.1% alpha-amylase (Sigma-Aldrich) for 10 min at 30°C and the other half were not. Then, glycogen was stained with PA-SH as described in [30], except that they were not dehydrated and cleared in xylol. Samples were mounted with Vectashield (Vector laboratories, Burlingame, USA) and analyzed with a Leica TCS SP2 confocal microscope.

### Transmission Electron Microscopy

Cells cultured on 10 cm-diameter dishes were fixed with 2.5% glutaraldehyde in 0.1 M phosphate buffer (PB) (pH 7.2) for 1 h and gently rocked at room temperature. Cell monolayers were scraped into 2 ml of PB. Then, they were washed with the same buffer and postfixed with 1% osmium tetroxide in the same buffer at 4°C for 1 h. The samples were dehydrated in acetone, infiltrated with Epon resin for 2 days, embedded in the same resin and polymerized at 60°C for 48 h. Ultrathin sections were obtained using a Leica Ultracut UCT ultramicrotome and mounted on Formvar-coated copper grids. They were stained with 2% uranyl acetate in water and lead citrate. Then, sections were examined by a JEM-1010 electron microscope (Jeol, Japan). The diameter and number of glycogen particles were measured by *analySIS *software (Olympus *Soft Imaging System*, Center Valley, USA).

### Western blotting

Aliquots of myotube or 293 cell extracts prepared for enzyme activity measurement were added with loading buffer consisting of 50 mM Tris-HCl (pH 6.8), 10 mM dithiothreitol, 2% SDS, 0.1% bromophenol blue and 10% glycerol. C2C12 and 293 cell extracts were prepared by scraping cell monolayers from 6 cm-diameter dishes into 100 μ1 of homogenization buffer consisting of 50 mM Tris-HCl (pH 7.5), 150 mM NaCl, 1 mM EDTA, 1 mM PMSF, 1 mM NaF, 1 mM Na_3_VO_4_, 2 μg/μl benzamidine, 2 μg/μl leupeptin, 1% Nonidet P40 and 1 mM dithiothreitol. Lysates were then gently rocked for 60 min at 4°C. An aliquot of the total cell extracts was used to measure protein concentration with the Pierce BCA protein assay kit. Protein was resolved by SDS/10%-PAGE, and immunoblotting was performed with antibodies against human PP1α, human PPP1R6 or mouse PTG (Santa Cruz Biotechnology Inc., Santa Cruz, USA), muscle GS or liver/muscle phospho-GS (Ser-641/0) (Cell Signalling, Danvers, USA), GFP (BD Biosciences, Palo Alto, USA) or human PPP1R3A (Sigma-Aldrich).

### Fusion construct R6-EGFP

A construct containing human PPP1R6 with an EGFP tag at the C terminus was prepared. To do this, a 910 pb fragment of the PPP1R6 cDNA was isolated from clone *IRAKp961M1391Q2 *by performing PCR with *Pfu *DNA polymerase. The sequences 5'CCGCGGTACCGTTCCGATGAAGTGGATCCAGCTCTCTTC3'and 5'AGAGAAGCTTATGTCCAGAGGCCCGAGCTCC3' were used, as downstream and upstream oligonucleotides respectively. Amplification conditions were 3 min at 94°C, followed by 45 cycles of 1 min at 94°C, 1 min at 65°C and 1 min at 72°C. A final extension was carried out at 72°C for 10 min. PCR products were purified by 1% agarose gel. The PCR-amplified fragment was 3' A-tailed by incubation with *Taq *DNA Polymerase in a reaction mixture containing MgCl_2 _and dATP, for 15 min at 70°C, and then cloned into the pGEM-T vector (Promega, Madison, USA) to generate pGEM-T-R6. Finally, pR6-EGFP was constructed by cloning the HindIII-KpnI PPP1R6 cDNA excised band from pGEM-T-R6 into the dephosphorylated pEGFP-N1 vector digested with the same restriction enzymes.

To cytolocate the R6-EGFP construct with the mitochondrial network, we used C2C12 mtRFP cells. Cells grown on coverslips were transfected with 5 μg of pR6-EGFP with the aid of GeneJuice (Merck Chemicals, Darmstadt Germany) and at 48 h post-transfection were fixed for 15 min with 4% paraformaldehyde in PBS. To cytolocate the R6-EGFP construct with the Golgi complex, C2C12 cells were cotransfected with 3 μg pR6-EGFP and 3 μg pRFP1-N1-GalT construct, which encodes RFP with the Golgi targeting domain of β-1,4-galactosyltransferase (GalTase) [31] (kindly provided by Jennifer Lippincott-Schwartz of NIH, Bethesda, MD, USA). pEGFP-N1 (BD Biosciences, San Jose, USA) was used to express EGFP. Cell preparations were mounted with Vectashield or ProLong Gold (Invitrogen, Paisley, UK) and analyzed with a Leica TCS SP2 confocal microscope.

### Statistical analysis

All data are given as means ± SEM, and the significance of the difference was analyzed by the Student's t test. Values were considered significant at p < 0.05. Results for gene expression (fold change from the real-time PCR analysis) were examined with REST (Relative Expression Software Tool).

## Results

### Relative expression of the PPP1R6 gene in human cultured myotubes and skeletal muscle tissue

The mRNA levels of PPP1R6 were analyzed in human skeletal muscle biopsy samples and in myotubes isolated from biopsies. In muscle biopsies, relative PPP1R6 mRNA levels (1/2^-ΔCt^) were lower than levels of the β2-microglobulin control gene (-19.7 ± 6.0). PPP1R6 gene expression in cultured myotubes (-453 ± 49) was 28.9 times less (p < 0.01) than in the biopsy tissue samples. In cultured myotubes, no differences were observed in PPP1R6 gene expression between cells cultured with glucose or 16 h glucose-deprived cells (data not shown).

### Effect of PPP1R6 overexpression on GS and GP activities

**Figure 1:**
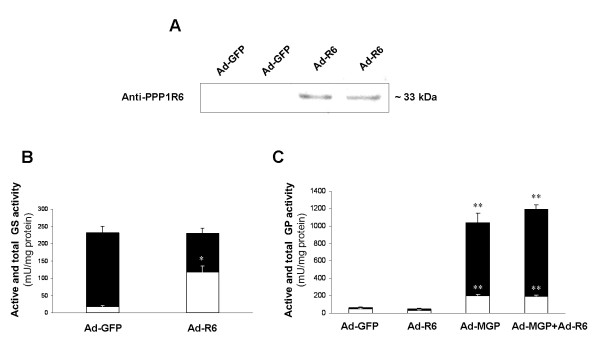
**Effect of PPP1R6 overexpression on GS and GP activity**. Cultured human myotubes were transduced with Ad-GFP, Ad-R6 or/and Ad-MGP and incubated with 25 mM glucose for 48 h. (A) Immunoblot analyses were performed on extracts (20 μg protein) from cells transduced with Ad-GFP or Ad-R6. Membranes were hybridized with antibody against PPP1R6. Enzyme activities were assessed in cell extracts: (B) GS activity measured with (black columns, total activity) or without (white columns, activity of the active form) glucose 6-phosphate and (C) GP activity measured with (black columns, total activity) or without (white columns, activity of the active form) AMP. Data are means ± SEM from two experiments performed in triplicate. Significance of differences versus cells treated with Ad-GFP under the same incubation conditions: *p < 0.001 and **p < 0.0001.

Cultured human myotubes were treated with the Ad-R6 adenovirus to overexpress human PPP1R6. Human PPP1R6 transcript levels were increased by a 2^-ΔΔCp ^factor of 2167 ± 217 (p = 0.001) in Ad-R6-treated cells, as assessed by RT and real-time PCR, compared to cells treated with the Ad-GFP virus and relative to the 18S rRNA control gene. In an immunoblot analysis the antibody against human PPP1R6 protein detected a band in Ad-R6-treated cells of about 33 kDa (Figure 1A), which was not shown in cells treated with the Ad-GFP virus, presumably due to a much lower PPP1R6 protein content.

The activity of the active form of GS in cell extracts increased 6 times more in PPP1R6-overexpressing cells than in cells treated with Ad-GFP, whereas total GS activity was unchanged (Figure 1B). In contrast, no effects of PPP1R6 overexpression were observed on GP activity, either the active form or total activity (Figure 1C). In the cultured myotubes the brain and liver isoforms of GP are greater contributors to GP activity, than the muscle GP isoform, as shown elsewhere [32], due to the drop in *PYGM *expression [22]. Thus, we tested whether PPP1R6 affected the activity of the muscle isoform of GP activity by transducing cells with an adenovirus encoding the muscle isoform of GP (Ad-MGP). In Ad-MGP-treated cells, the activity of the active form of GP and total GP activity increased 4 and 16 times, respectively; neither of them was affected by PPP1R6 delivery (Figure 1C).

### Differential regulating effects of PPP1R6, PTG and G_M _on GS and glycogen synthesis

We further investigated the activating effect of PPP1R6 on GS and the impact on glycogen accumulation versus the PP1-GTSs, PTG and G_M_. Thus, cultured muscle cells were transduced with Ad-PTG virus and PTG expression was confirmed by immunoblot analysis showing an immunoreactive protein band (of about 36 kDa) not detected in Ad-GFP-transduced cells, as in [18] (data not shown). G_M _overexpression was achieved by transduction with the Ad-G_M _virus, which increased the mRNA level of rabbit G_M _by a factor of 412 ± 34 (p = 0.001) over cells treated with Ad-GFP and relative to the 18S rRNA control gene, as assessed by RT real-time PCR. Immunoblot analysis showed an immunoreactive protein band (of about 124 kDa) not detected in Ad-GFP-transduced cells, as in [16] (data not shown).

**Figure 2:**
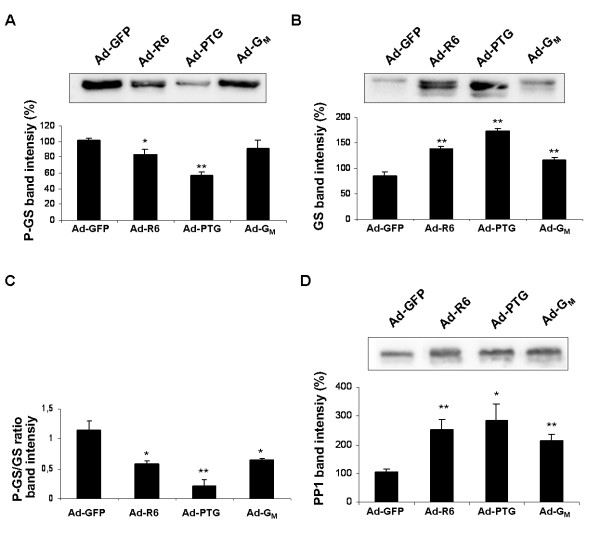
**Immunoblot analysis of GS and PP1 protein content**. Cultured human myotubes were transduced with Ad-GFP, Ad-R6, Ad-PTG or Ad-G_M _and incubated with 25 mM glucose for 48 h. Immunoblot analyses were performed on cell extracts (20 μg protein for GS and phospho-GS and 5 μg protein for PP1). Membranes were hybridized with antibodies against: (A) phospho-GS (Ser-641/0), (B) muscle GS and (D) PP1α. (A, B, D) A representative image is shown. Bands were quantified with a LAS-3000 (FujiFilm). Data are means ± SEM from three experiments performed in duplicate. Significance of differences versus cells treated with Ad-GFP under the same incubation conditions: *p < 0.05 and **p < 0.01.

We analyzed the phospho-GS (Ser-641/0), total muscle GS and PP1α protein content in cell extracts (Figure 2). The phospho-GS (Ser-641/0) protein level was slightly reduced (18%) by PPP1R6 overexpression, compared to the phosphorylation degree in Ad-GFP-treated cells, whereas PTG caused a striking reduction (44%) and G_M _caused a reduction that did not reach statistical significance. In accordance with these observations, an immunoblot analysis of muscle GS protein showed a downward shift in the electrophoretic mobility, indicating dephosphorylation of the enzyme, in response to PTG and PPP1R6. Total muscle GS protein content was increased by the PP1-GTSs in the order of magnitude: PTG > PPP1R6 > G_M_. In consequence, the phospho-GS/total muscle GS protein ratio was significantly reduced by the three PP1-GTSs (49% PPP1R6, 81% PTG and 43% G_M_). Finally, the total protein content of PP1α, whose transcript in human tissues is most abundant in skeletal muscle and heart [33], was also increased by PTG, PPP1R6 and G_M _in the same order of magnitude as total muscle GS protein was.

**Figure 3:**
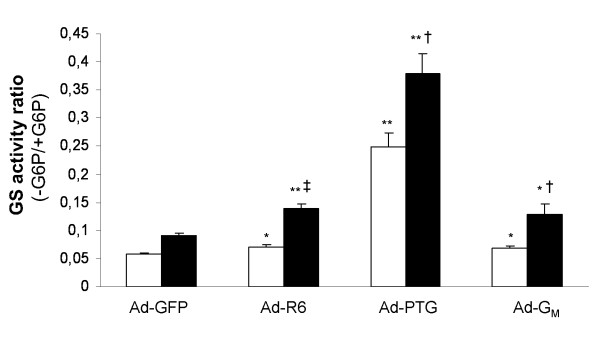
**GS activity ratio in glucose-replete and -depleted cells**. Cultured human myotubes were transduced with Ad-GFP, Ad-R6, Ad-PTG or Ad-G_M _and then incubated with (white columns) or without (black columns) 25 mM glucose for 24 h. GS activity ratio (without glucose 6-phosphate/with glucose 6-phosphate) was measured in cell extracts. Data are means ± SEM from five experiments performed in duplicate. Significance of differences: versus cells treated with Ad-GFP under the same incubation conditions, *p < 0.05 and **p < 0.0001; cells without glucose versus with glucose for any viral treatment, †p < 0.01 and ‡p < 0.0001.

Next we examined the glucose-dependence of activating effects on GS activity of the three PP1-GTSs (Figure 3). Delivery of PPP1R6 enhanced GS activity ratio in both glucose-replete (23%) and -depleted (56%) cells versus Ad-GFP-cells, although the effect was stronger under glucose depletion. Similar glucose dependence was observed for G_M _in accordance with our previous data [16]: 17% versus 44% in glucose-replete and -depleted cells, respectively. In contrast, PTG strongly activated GS, irrespective of glucose presence (4.4-fold) or absence (4.2-fold), as we showed elsewhere [18].

**Figure 4:**
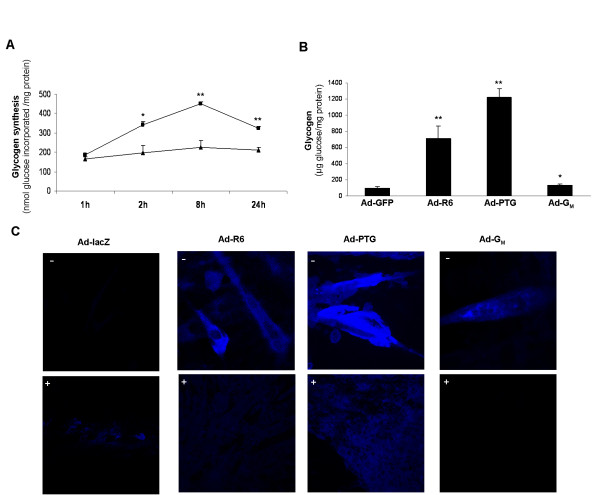
**Glycogen synthesis and content**. Cultured human myotubes were: (A) transduced with Ad-GFP (▲) or Ad-R6 (■) and incubated with glucose-deprived medium for 18 h, then with 10 mM [U-^14^C]glucose for the times indicated and harvested to quantify the radioactivity associated with glycogen; (B) transduced with Ad-GFP, Ad-R6, Ad-PTG or Ad-G_M_, incubated with 25 mM glucose for 48 h and harvested to assess glycogen content. (A, B) Data are means ± SEM from three experiments performed in duplicate. Significance of differences versus cells treated with Ad-GFP: *p < 0.05 and **p < 0.001. (C) C2C12 cells were transduced with Ad-lacZ, Ad-R6, Ad-PTG or Ad-G_M _and incubated with 25 mM glucose for 72 h. Fixed cell monolayers were treated without (-) or with (+) alpha-amylase and glycogen stained with fluorescent PA-SH. A representative image is shown.

The effect of PPP1R6 on glycogen synthesis rate was estimated by the incorporation of radioactively labeled glucose into glycogen after a period of glucose deprivation. A time-course study showed that, in cells treated with the control Ad-GFP virus, glucose incorporation was steady over time, but that in cells overexpressing PPP1R6 there was progressive enhancement of glucose deposition into glycogen during the first 8 h, whereas at 24 h the increase was smaller (Figure 4A). Glycogen content, as assessed after KOH-extraction, ethanol precipitation, hydrolysis with alpha-amyloglucosidase and quantification of released glucose, was increased by PPP1R6 overexpression (7-fold) (Figure 4B) much less than glycogen accumulated in response to PTG overexpression (12-fold increment), but more than it was by G_M _overexpression (1.4-fold increment).

Glycogen was then revealed cytochemically as blue fluorescent PA-SH stained material in fluorescence microscopy (Figure 4C). In PPP1R6-overexpressing cells, diffuse cytoplasmic fluorescence was observed at a much higher intensity than in cells transduced with the control virus Ad-lacZ. Fluorescent intensity was, however, much higher in cells transduced with Ad-PTG, whereas a fainter signal was detected in cells transduced with Ad-G_M_. The PA-SH fluorescently stained material was drastically removed by digestion with alpha-amylase in all PP1-GTS-cells, indicating that it was due to glycogen presence.

**Figure 5:**
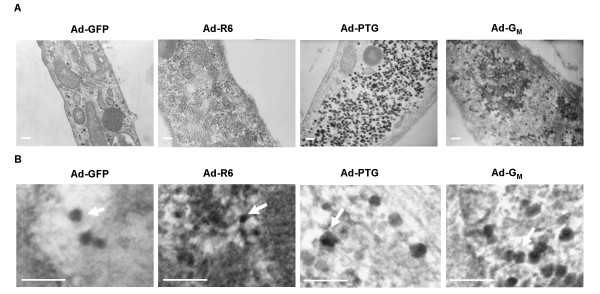
**Glycogen particles**. Cultured human myotubes were transduced with Ad-GFP, Ad-R6, Ad-PTG or Ad-G_M _and incubated with 25 mM glucose for 48 h and then fixed and stained for observation of glycogen particles in transmission electronic microscopy. Representative images are shown: (A) image of myotubes obtained at × 40000 magnification, in which glycogen granules appear as dense spheroid particles, and (B) a detail of glycogen granules (arrows). Scale bars = 100 nm.

**Figure 6:**
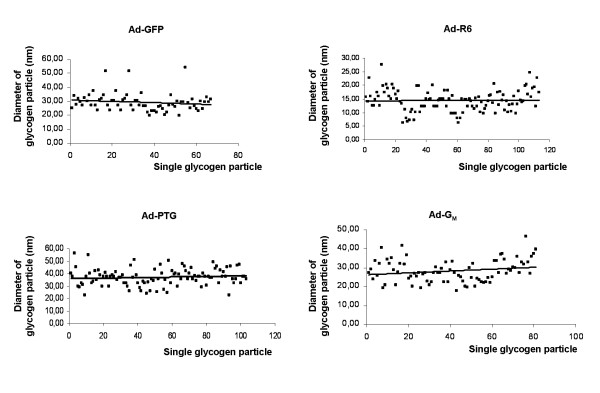
**Diameter of glycogen particle**. The graphics plot the diameter of each measured glycogen granule (at least 70 quoted granules for each condition) in cultured human myotubes transduced with Ad-GFP, Ad-R6, Ad-PTG or Ad-G_M _and incubated with 25 mM glucose for 48 h.

Glycogen granules were examined in myotube sections by transmission electron microscopy (Figure 5). In control Ad-GFP-cells, glycogen was revealed as spherical dense particles (Figure 5A and 5B) scattered through the cytosol (Figure 5A). The number of particles counted in control cells was 39 ± 12 particles/μm^2^. The mean diameter of particles was 29.2 ± 0.7 nm and the majority of particles were within 20 to 30 nm although some of them reached more than 50 nm, as shown in Figure 6. In PPP1R6-overexpressing cells, there were many more dense round particles (288 ± 20 particles/μm^2^, p < 0.0001), whose mean diameter 14.4 ± 0.4 nm (p < 0.0001) was smaller, while the range was from 7 to 24 nm. These PPP1R6-derived particles appeared as membrane-, vesicle- or organelle-associated granules. In PTG-cells a marked increment in the number of particles was also seen (202 ± 38 particles/μm^2^, p < 0.005), but particles had a much larger diameter of 36.9 ± 0.7 nm (p < 0.0001) and sizes ranged from higher than 23 to more than 50 nm. These were located in the cytosol in large areas filled with glycogen particles, which appeared devoid of organelles and not surrounded by a membrane. Finally, G_M_-overexpression caused a smaller rise in the number of glycogen particles (75 ± 7 particles/μm^2^, p < 0.05), which were concentrated in clusters close to certain organelles, notably the endoplasmic reticulum, and the subsarcolemma. The average size of G_M_-derived glycogen granules was 28.3 ± 0.7 nm and diameters ranged from 17 to 45 nm.

### Intracellular location of the fusion protein R6-EGFP

We analyzed by computational methods the possible intracellular location of PPP1R6. The Hum-mPLoc [34] server that predicts subcellular location at multiple locations predicted Golgi membrane location for PPP1R6, while the Golgi predictor [35] server implemented for Type II transmembrane proteins gave a 26.11 index (threshold 20.00) for Golgi location. However, the TargetP 1.1 [36] server, which also predicts the subcellular location of eukaryotic proteins, gave a 0.695 probability that the sequence contains a mitochondrial targeting peptide, while the MitoProtII server [37], which predicts mitochondrially-imported proteins, gave a 0.8578 probability that human PPP1R6 protein would be transported to mitochondria.

**Figure 7:**
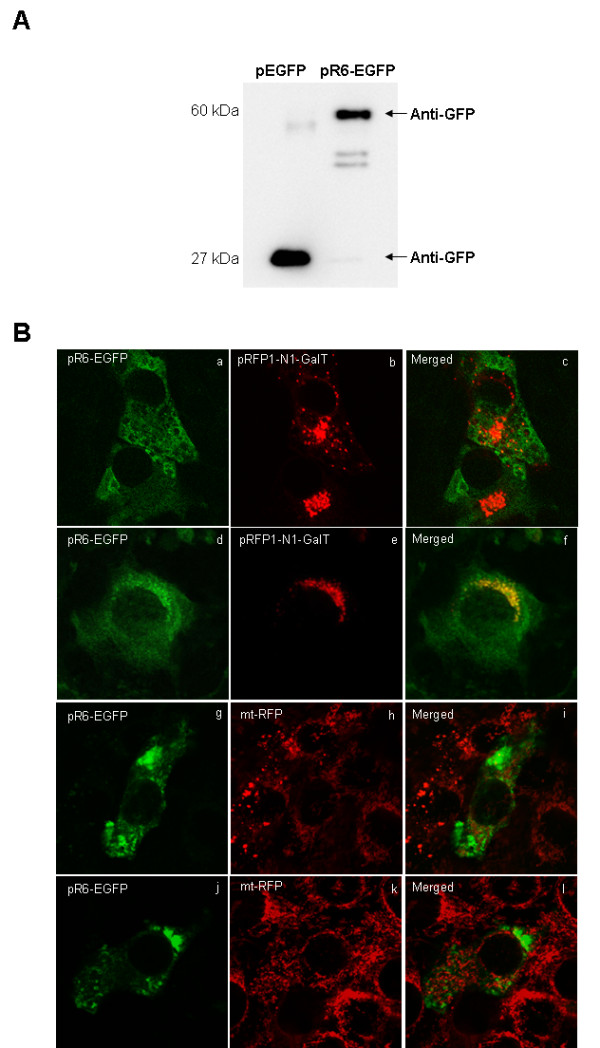
**Cytolocation of R6-EGFP**. (A) 293 cells were transfected with 5 μg of plasmids encoding EGFP or R6-EGFP with the aid of GeneJuice, at 48 h post-transfection a Western blotting analysis was performed on cell extracts (30 μg protein) and membranes were hybridized with an antibody against GFP. (B) (a to f) C2C12 myoblasts were cotransfected with pR6-EGFP and pRFP1-N1-GalT, at 48 h post-transfection, cells were incubated with 25 mM glucose (a to c) or without glucose (d to f) for 16 h and a colocation analysis of EGFP and RFP was performed; (g to l) C2C12-mtRFP myoblasts were transfected with pR6-EGFP, at 48 h post-transfection, cells were incubated with 25 mM glucose (g to i) or without glucose (j to l) for 16 h and then colocation analysis of EGFP and RFP was performed. The image shows the fluorescent signal of EGFP (a, d, g, j) and RFP (b, e, h, k) and the colocation of the signal of EGFP and RFP (c, f, i, l).

Then, we constructed a fusion protein of PPP1R6 with a C-terminal EGFP flag (R6-EGFP). Expression of the fusion protein and its glycogenic activity was confirmed after transfection of the plasmid encoding R6-EGFP into 293 cells. Immunoblot analysis with anti-PPP1R6 antibody showed a band of about 60 kDa molecular weight, higher than the endogenous PPP1R6, in cells transfected with R6-EGFP and not in those transfected with EGFP (data not shown). A band of this same size was also recognized by the anti-GFP antibody in R6-EGFP cells, whereas a band of about 27 kDa was revealed in EGFP-cells (Figure 7A). In 293 cells expressing the R6-EGFP construct glycogen synthesis (120 ± 2 nmol glucose incorporated/mg protein) was higher (p < 0.0001) than in EGFP-cells (55 ± 0.4 nmol glucose incorporated/mg protein).

To assess whether PPP1R6 was located at the Golgi complex, we cotransfected R6-EGFP and the Golgi marker, RFP1-N1-GalT, into C2C12 myoblasts incubated with and without glucose. As shown in Figure 7B, in glucose-replete cells, R6-EGFP appeared throughout the cytosol with a diffuse pattern and no colocation with RFP1-N1-GalT was found. In glucose-depleted cells, R6-EGFP showed a similar cytosol pattern plus a punctuated pattern clustered around the nucleus that was colocated with the Golgi marker. The Golgi marker RFP1-N1-GalT displayed a compact structure in the perinuclear region mainly covering one of the nucleus poles in glucose-replete cells and appeared in punctuate structures surrounding the nucleus in glucose-depleted cells, according to the fact that ATP depletion induces the Golgi apparatus disassembly [38]. To examine whether PPP1R6 was located at the mitochondria, R6-EGFP was delivered to C2C12 mtRFP myoblasts, which express a mitochondrial matrix-targeted RFP (Figure 7B). No colocation of R6-EGFP was observed with mt-RFP either in glucose-replete or -depleted cells. As a control, we transfected C2C12 and C2C12 mtRFP myoblasts with a plasmid encoding EGFP and we observed that this protein was located both in the cytoplasm and nucleus of glucose-replete or -depleted cells and that it was not colocated with either the mitochondrial matrix-targeted or Golgi-targeted RFP (data not shown).

## Discussion

PPP1R6 was originally described as a glycogen-associated PP1 regulatory subunit with wide tissue expression and most common in humans in skeletal muscle and heart [9]. Here we report the lower expression of the PPP1R6 gene in human cultured myotubes than in skeletal muscle tissue. In this regard, PPP1R6 resembles PPP1R3C and differs from the PPP1R3B gene, whose expression is maintained in culture, and also from the muscle-specific PPP1R3A gene, whose expression is almost suppressed [19]. Therefore, on the basis of our data from this and the earlier study [19], the relative differential gene expression is PPP1R3C > PPP1R3A > PPP1R6 > PPP1R3B in human skeletal muscle tissue and PPP1R3C > PPP1R3B > PPP1R6 > PPP1R3A in cultured human myotubes.

In cultured myotubes, PPP1R6 activated GS and reduced its phosphorylation at Ser-641/0, also referred to as site 3a, in a less powerful manner than PTG, but stronger than G_M_. GS may be phosphorylated at nine or more sites [39], but the Ser-641/0 site is crucial for the regulation of GS activity and is extensively regulated by various effectors [4]. The selectivity of PP1 for the different sites of GS phosphorylation has not been established, but it has been proposed that PP1-GTSs may confer such selectivity [4]. Our data provide evidence that the degree of dephosphorylation of the Ser-641/0 site achieved in response to PPP1R6, PTG or G_M _is tightly associated with that of GS activation, thereby suggesting a similar strong control of enzyme activity through the modulation of the phosphorylation of this site by these PP1-GTSs. The activating effect of PPP1R6 on GS was enhanced by glucose/glycogen depletion, as also observed for G_M _[16] but not PTG [18]. In fact, glycogen content is a strong regulator of GS and high glycogen content impairs insulin-stimulated activation of this enzyme, as reviewed in [4]. PPP1R6 did not exert any effect on the cultured myotube GP activity, either the endogenously expressed isoforms, which are predominantly the brain (PYGB) and liver (PYGL) isoforms [32], or the overexpressed muscle (PYGM) isoform. Like PPP1R6, neither does PTG affect endogenous myotube GP activity [18], whereas G_M _inactivates GP [16]. As PPP1R6 markedly activated GS in myotubes, it exerted a potent glycogenic effect. This was seen by the increases in the rate of glycogen synthesis; by an increase in the net glycogen content, as quantified after KOH extraction and ethanol precipitation; and cytochemically, by an increase in the glycogen stained as blue fluorescent PA-SH material.

Glycogen accumulation in response to PPP1R6 was less than that achieved with PTG but higher than with G_M_. Cellular glycogen is complexed with proteins, including glycogen metabolizing enzymes, PP1 and PP1-GTSs [3], in the form of particles or granules, also named glycosomes [1,2]. The morphology of these granules was originally defined by Drochmans [40] as α-, β- and γ-types. The α particles, commonly found in mammalian liver, are agglomerates of β-particles, have a rosette appearance and large diameters [1], in the order of 100 nm [40]. Single β-particles are found in most normal tissues, including skeletal muscle [1], have a spheroid shape and diameters ranging from 15 to 40 nm. The *γ*-particles are about 3 nm in diameter and are subunits of β- and α-particles [1]. Glycogen particle sizes in skeletal muscle cells have been consistently shown to follow a continuous distribution pattern. In human skeletal muscle, diameters range from 10 to 44 nm and a mean value of 25 nm at rest [41] and from 8 to 43 nm and a mean particle size of about 13 nm after exercise [42]. In cultured rat myotubes, these particles range from above 15 to above 40 nm (mean value of 29.4 nm) in glycogen-replete cells or from above 10 to below 40 nm (mean value of 24.9 nm) in glycogen-depleted ones [43]. Strikingly, here we show that, despite being in a continuous range, the size distribution pattern of glycogen particles is determined by PP1-GTS scaffolding, with average diameters in the order of PPP1R6 < G_M _< PTG. In control myotubes most glycogen particles corresponded to the β-type, with a mean diameter of 29.2 nm. No particles smaller than about 20 nm were observed, but a few above 42 nm were found. Notably, the detection limit for small glycogen granules is estimated to be 5-10 nm (at 20000 × magnification) [42], while the predicted maximum size limit of a granule is 42 nm, consisting of 12 tiers of carbohydrate [3]. PPP1R6 generated small granules, with a mean diameter of 14.4 nm, with most of the granules falling below 20 nm and many between 7 and 15 nm. These granules resembled the γ-type in the formation of filamentous alignments [40]. G_M_-derived glycogen particles showed an intermediate mean diameter of 28.3 nm and most were between 20 and 40 nm, which corresponds to the β-particle classification. PTG produced the largest particles, with a mean diameter of 36.9 nm and values ranging from 25 to 50 nm. Many of these particles looked like β-type ones, but we observed a few supramolecular clusters with a rosette-like shape that resembled α-particles. In fact, the larger α-type glycogen particles have not been observed in mammalian tissues other than liver [40]. Since PTG is expressed abundantly in liver, in rats [44] and humans [9], we speculate that it induces the formation of α-particles in this tissue. In this regard, β particles are suggested to be covalently bonded to form α-particles through a hitherto unsuspected enzyme process operative in the liver [45], which could be tissue-dependent.

The number of glycogen particles was increased by the three PP1-GTSs tested. PPP1R6 and PTG induced the greatest increases in particle numbers and quantified glycogen accumulation. The rise in particle numbers per cell area for PPP1R6 was higher than with PTG, although quantification of extracted or cytochemically stained glycogen was not greater, probably because PTG-derived particles have bigger size and tend to form agglomerations of single β-particles. G_M _caused the smallest increase in the number of particles per cell area, which fits with glycogen quantification data. In fact, early muscle glycogen resynthesis has been shown to also rely on the synthesis of new particles. Elsner et al. [43] suggested that during early glycogen resynthesis in fasted cultured myotubes, new glycogen molecules are formed while there is a modest increase in glycogen particle diameter. Graham and coworkers [42] showed that, during recovery from prolonged exercise, resynthesis of muscle glycogen in humans is characterized initially by an increase in number and no change in particle size and later by an increase in particle size but not in number.

The subcellular distribution of glycogen-particles promoted by either PP1-GTS has distinct patterns. In PPP1R6-myotubes, granules were closely associated with cellular components. In G_M_-myotubes, granules accumulated near the endoplasmic reticulum, which is consistent with G_M _association with SR via an SR-binding sequence in its C-terminal region [8,20,21]; however, granules also appeared in other locations, preferentially subsarcolemmal ones. In fact, two forms of G_M_, an SR-associated form and a cytosolic glycogen-associated form, can be distinguished in rat skeletal muscle extracts [46]. In contrast, PTG caused the accumulation of glycogen granules in large clusters located in central areas of the cytosol devoid of cellular organelles. In control cells, glycogen granules were distributed throughout the cytosol. Since in control myotubes the PPP1R3C gene is the most expressed gene (of PPP1R3A, PPP1R3B, PPP1R3C and PPP1R6 genes) and PTG is strongly glycogenic, it can be argued that glycogen particles in these cells are mainly PTG-derived, which may explain their preferential cytosolic location. Smaller granules were observed in control myotubes than in PTG-overexpressing cells, probably due to much lower glycogen accumulation in the former. We cannot rule out, however, a role for the still unstudied PPP1R3E protein. In the skeletal muscle tissue of humans [3,41,47,48] and rats [49], electron microscopy studies have shown compartmental distribution of glycogen particles in three main regions: subsarcolemmal, intermyofibrillar and intramyofibrillar. In the subsarcolemmal location, clustered accumulations are observed at the level of mitochondria and SR [47], and nuclei and mitochondria [41]. Aneurally cultured human myotubes are not fully differentiated, and likely this influences the pattern of cellular glycogen distribution. However, with PPP1R6 and G_M _we observed clustering of glycogen granules to the membrane of myotube organelles.

Finally, here we applied computational scanning of the PPP1R6 protein sequence to gain insight into its potential subcellular distribution. These analyses revealed a high probability that PPP1R6 is located at the Golgi complex or imported into the mitochondria. We thus analyzed the subcellular distribution of PPP1R6 tagged at the C-terminus with EGFP. The tagged PPP1R6 showed a diffuse pattern in the cytosol in glucose-replete and -depleted cells and a punctuate pattern clustered around the nucleus in glucose-depleted cells only. Since no colocation of tagged PPP1R6 with the mitochondrial-targeted RFP was observed, irrespective of glucose incubation, no support for the mitochondrial export premise was obtained. Neither was colocation of the tagged PPP1R6 with the Golgi-targeted RFP in glucose-replete C2C12 myoblasts. However, in glucose-depleted myoblasts the two proteins overlapped in the perinuclear region, according to the sequence-based prediction of PPP1R6 location in the Golgi complex. Therefore, our data suggest that PPP1R6 translocates in response to glucose from the Golgi complex to a cytosolic location, where it is associated with glycogen. Along this line of argument, PPP1R6 present in the glycogen/sarcovesicle fraction isolated from rabbit skeletal muscle is specifically associated with glycogen, since it is released by digestion of glycogen with α-amylase [9]. The role that PPP1R6, or PPP1R6-targeted PP1, may exert in the Golgi apparatus in glucose-depleted myotubes is beyond the scope of this study. For instance, PP1 activity was previously reported to be essential in regulating vacuolar fusion and endoplasmic reticulum-to-Golgi and endocytic vesicular trafficking in the yeast *Saccharomyces cerevisiae *[50]. Noteworthy, in muscle cells GS also shows differential intracellular distribution as a function of glycogen content. In skeletal muscle, GS translocates from a glycogen-enriched membrane fraction to the cytoskeleton as glycogen content is lowered [51] or it is found associated with spherical structures formed by actin cytoskeleton rearrangement after glycogen-depletion [52,53]. In cultured C2C12 cells, muscle GS concentrates in the nucleus in the absence of glucose and translocates to the cytosol in response to glucose [54]. These data suggest that after cellular glycogen depletion, some glycogen-associated proteins, such as PPP1R6 and GS, may translocate to diverse cell compartments.

## Conclusions

PPP1R6 gene expression has been described as highest in skeletal muscle and heart; however its relative transcript levels are much lower than those of other PP1-GTSs in both human skeletal muscle tissue and primary cultured muscle. Although PPP1R6 has low protein sequence homology with human G_M _and PPP1R5 proteins [9], it exerts a powerful glycogenic effect in cultured muscle cells, more than G_M _and less than PTG. PPP1R6 translocates in response to glucose from the Golgi complex to the cytosol, where it is presumably associated with glycogen. Finally, we show that the pattern of size and subcellular distribution of glycogen particles is differentially determined by PPP1R6, PTG or G_M _used as molecular scaffolding, which reveals that PP1-GTSs confer glycogen granule regulation.

## Abbreviations

PP1: protein phosphatase 1; PP1-GTS: PP1 glycogen-targeting subunit; PP1c: PP1 catalytic subunit; GS: glycogen synthase; GP: glycogen phosphorylase; SR: sarcoplasmic reticulum; mt: mitochondrial matrix-targeted; RFP: red fluorescent protein; EGFP: enhanced green fluorescent protein; FBS: fetal bovine serum; HS: horse serum; RT: reverse transcription; Ct: threshold cycle; Cp: crossing point; Ad-GFP: an adenovirus encoding EGFP; Ad-lacZ: an adenovirus encoding the *E. coli *ß-galactosidase gene with a nuclear location signal; Ad-R6: an adenovirus encoding human PPP1R6; Ad-G_M_: an adenovirus encoding rabbit G_M; _Ad-PTG: an adenovirus encoding mouse PTG; Ad-MGP: an adenovirus encoding rabbit muscle GP; PA-SH: periodic acid/salicyloyl hydrazide; PB: phosphate buffer; R6-EGFP: a fusion protein of PPP1R6 with a C-terminal EGFP flag.

## Authors' contributions

MM carried out the gene expression analysis, construction of Ad-R6 and transduction with recombinant adenoviruses, performed the metabolic experiments, measure of enzyme activities, electron microscopy analysis, western blotting, cytolocation of R6-EGFP construct with the Golgi complex, the statistical analysis, figures and participated in the manuscript drafting. MG carried out the preparation of pR6-EGFP construct and performed the analysis of cytolocation of R6-EGFP with the mitochondrial network. AO carried out the cell growth and participated in the electron microscopy analysis. CG participated in the construction of Ad-R6^.^AMG conceived the study, and participated in its design and coordination and drafted the manuscript. All authors read and approved the final manuscript.
